# Exploration of the shared pathways and common biomarker PAN3 in ankylosing spondylitis and ulcerative colitis using integrated bioinformatics analysis

**DOI:** 10.3389/fimmu.2023.1089622

**Published:** 2023-01-18

**Authors:** Minna Zhang, Junyi Zhou, Honggang Wang, Le He, Jingyi Wang, Xiaozhong Yang, Xiaomin Zhong

**Affiliations:** ^1^Department of Gastroenterology, The Affiliated Huai’an No.1 People’s Hospital of Nanjing Medical University, Huai’an, Jiangsu, China; ^2^Department of Oncology, The Huai’an Clinical College of Xuzhou Medical University, Huai’an, Jiangsu, China; ^3^Digestive Disease Center, The Affiliated Huai’an No.1 People’s Hospital of Nanjing Medical University, Huai’an, Jiangsu, China

**Keywords:** ulcerative colitis, ankylosing spondylitis, weighted gene co-expression network analysis, mitogen-activated protein kinase, *PAN3*

## Abstract

**Background:**

Ulcerative colitis (UC) is a chronic autoimmune-related disease that causes inflammation of the intestine. Ankylosing spondylitis (AS) is a common extraintestinal complication of UC involving the sacroiliac joint. However, the pathogenesis of AS secondary to UC has not been studied. This study aimed to investigate the shared pathways and potential common biomarkers of UC and AS.

**Methods:**

Microarray data downloaded from the Gene Expression Omnibus (GEO) database were used to screen differentially expressed genes (DEGs) in the UC and AS datasets. Weighted gene co-expression network analysis (WGCNA) was performed to identify co-expression modules related to UC and AS. Shared genes were then further analyzed for functional pathway enrichment. Next, the optimal common biomarker was selected using SVM-RFF and further validated using two independent GEO datasets. Finally, immune infiltration analysis was used to investigate the correlation of immune cell infiltration with common biomarkers in UC and AS.

**Results:**

A total of 4428 and 2438 DEGs in UC and AS, respectively, were screened. Four modules were identified as significant for UC and AS using WGCNA. A total of 25 genes overlapped with the strongest positive and negative modules of UC and AS. KEGG analysis showed these genes may be involved in the mitogen-activated protein kinase (MAPK) signaling pathway. GO analysis indicated that these genes were significantly enriched for RNA localization. *PAN3* was selected as the optimal common biomarker for UC and AS. Immune infiltration analysis showed that the expression of *PAN3* was correlated with changes in immune cells.

**Conclusion:**

This study first explored the common pathways and genetic diagnostic markers involved in UC and AS using bioinformatic analysis. Results suggest that the MAPK signaling pathway may be associated with both pathogeneses and that *PAN3* may be a potential diagnostic marker for patients with UC complicated by AS.

## 1 Introduction

Ulcerative colitis (UC) is a chronic autoimmune-related inflammatory disease with symptoms occurring both inside and outside the gastrointestinal tract (1). The incidence of UC is increasing annually with changes in economic growth, diet, and lifestyle habits (2). Clinically, UC is characterized by recurrent diarrhea, bloody purulent stools, and abdominal pain (3). Some patients also have extraintestinal manifestations (EIM), with primary involvement of the musculoskeletal system, skin, hepatobiliary tract, and eyes, which seriously affects the patient’s quality of life. However, the pathogenesis of EIM is poorly understood. It has been suggested that EIM may be linked to intestinal immune response dysfunction (4). Additionally, it is thought EIM may be an independent inflammatory event triggered by the persistence of inflammatory bowel disease (IBD) (5). Moreover, environmental, and genetic factors may also contribute to the progression (5). Ankylosing spondylitis (AS) is a common autoimmune disease characterized by inflammation of the sacroiliac joints and spinal attachments (6). A German study suggested that 5–10% of AS is associated with IBD, and researchers found a greater proportion of AS patients had subclinical bowel inflammation (7). Consistently, inflammatory arthropathy is the most common extraintestinal complication in IBD patients, with a prevalence of 20%-50% for axial inflammation (8), and 5%-20% for peripheral arthritis (4). The prevalence of AS in patients with IBD is estimated to be 3% (9). Human leukocyte antigen B27 (HLA-B27) positivity is associated with idiopathic AS in over 90% of cases; however, HLA-B27 is less specific and sensitive in spondylitis complicated by IBD (10). Moreover, the progression of AS, from inflammatory back pain to radiographic sacroiliitis often takes years to develop (11). Therefore, finding more specific diagnostic markers for IBD patients suffering from back pain, and carrying out earlier treatment interventions for IBD patients with AS is of great clinical significance. In this study, published gene expression data in the GEO database was reviewed. Shared gene pathways and diagnostic markers between UC and AS were then explored using a systems biology approach. Using this data, we aimed to determine new potential diagnostic and treatment strategies for patients with AS secondary to UC.

## 2 Methods

### 2.1 Datasets and data pre-processing

We obtained raw gene expression profile data and clinical information from the GEO database. We searched the GEO database for RNA-seq profiles using the keywords “ulcerative colitis” and “ankylosing spondylitis”. The following filtering criteria were used: The tissue used for sequencing should be peripheral blood mononuclear cells (PBMCS), and the number of samples per group should not be less than 10 to ensure the accuracy of WGCNA.

Finally, The GEO datasets numbered GSE73754, GSE126124, GSE25101, and GSE3365 were obtained. The GSE73754 dataset was used on the GPL10558 platform. The dataset contained 72 samples, including 52 peripheral blood samples from AS patients and 20 from healthy controls. The GSE126124 dataset was used on the GPL6244 platform. This dataset contained 50 samples, including 18 peripheral blood samples from UC patients and 32 peripheral blood samples from healthy controls. Additionally, the GSE25101 dataset based on GPL6947 was regarded as an external validation set from the GEO database with 16 cases of AS patients as the experimental group and 16 cases of normal samples as the control group. Similarly, the GSE3365 dataset was also regarded AS the external validation set of the GEO database based on the GPL96 platform, with 26 UC patients as the experimental group and 42 normal samples as the control group (**Figure S1**).

### 2.2 Differential genetic screening

Differentially expressed genes (DEGs) were screened in GSE73754 and GSE126124 data sets. Limma R package was used to screen DEGs, and p. adj. value < 0.05 was selected as the cut-off standard.

### 2.3 Construction and module analysis of weighted gene co-expression network (WGCNA)

The WGCNA (Weighted Gene Co-expression Network Analysis) software package R.4.0.3 is used to perform WGCNA analysis. WGCNA R package was used to construct co-expression networks with corresponding clinical characteristics for DEGs of UC and AS. Prior to analysis, hierarchical cluster analysis was performed using the Hclust function in R language to exclude outlier samples. Then, according to the standard of scale-free network, the “pickSoftThreshold” function in the WGCNA software package is used to select the appropriate soft power β (ranging from 1 to 20) for automatic network construction. The results are clustered by topological overlap matrix analysis, which contains module assignments labeled by color and module feature (ME). In addition, Pearson’s correlation test was used to calculate the correlation between ME and clinical features. The modules with a |ME|> 0.3 and a p-value < 0.05 were considered in the interaction with clinical features is of great significance.

### 2.4 Enrichment analysis

Gene Ontology (GO) enrichment analysis is a commonly used bioinformatics method to search for comprehensive information on large-scale gene data. In addition, KEGG pathway enrichment analysis has been widely used to understand biological mechanisms and functions. The GOplot program package was used to visualize the GO and KEGG pathways. Finally, the clusterprofile package and GSVA package are used to further explore important signaling pathways. In addition, the gene sets and gene expression matrices of UC and AS were analyzed by GSEA to explore the possible regulatory pathways involved.

### 2.5 Identification and validation of potential shared diagnostic markers

Support vector machine recursive feature elimination is a machine learning method based on support vector machine, which finds the optimal core gene by deleting the feature vectors generated by support vector machine. In the intersection genes of DEGs and WCGNA mentioned above, SVM method was used to screen the core markers. Finally, the area under the ROC curve (AUC) was used to evaluate the diagnostic efficacy of core markers in the modeling set and validation set of UC and AS.

### 2.6 Extraction of peripheral blood mononuclear cell(PBMC)

We used purple anticoagulation tubes to collect 6 ml of blood samples from 3 UC patients, 3 AS patients, and 3 normal controls. I the ultra-clean table, human peripheral blood, and phosphate buffer saline (PBS) evenly were mixed in a ratio of 1:1 (6 ml: 6 ml). 3 ml of lymphocyte separation medium was added (BL590 Biosharp) into each 15 ml centrifuge tube and 6 ml of diluted blood was gently added on top of lymphocyte isolation medium. Then we placed the centrifuge tubes in the centrifuge for centrifugation for 20-25 min (400 g, 25 °C, ACCECL 2, DECELE 2).The cloud layer at the middle and upper interface was carefully collected and transferred to a new 15 mL tube. Finally, we washed PBMC with PBS, and discarded supernatant after centrifugation. Each patient signed the informed consent form in advance and approved by the Ethics Committee of the First People’s Hospital of Huai’an.

### 2.7 Quantitative real-time PCR (qRT-PCR)

Total RNA was extracted from Peripheral blood mononuclear cell (PBMC) by RNA simple total RNA kit (TIANGEN, DP419). 2 μL of total RNA was pipetted for measurement by Nanodrop. RNA was then transcribed into cDNA using a PrimeScript™ RT Master Mix kit (TAKARA, Ref. RR036A). qRT-PCR was performed using a LightCycler Instrument. PCR system using SYBR○Rpremix Ex Taq™ (TAKARA, RR420A) following manufacturer’s instructions. The relative mRNA levels were normalized to those of GAPDH using the 2-ΔΔCt method. (PAN-Fwd: 5’-GAAACTGTTGGTGGGACGACTTA-3’; PAN-Rev: 5’-TGAGGTGCAGTTGGAGGATAAAT-3’; GAPDH-Fwd: 5’-ATGGGGAAGGTGAAGGTCG-3’; GAPDH-Rev: 5’-CTCCACGACGTACTCAGCG-3’).

### 2.8 Immune analysis algorithm

The CIBERSORT algorithm calculates the proportion of different immune cell types based on the expression levels of immune cell-related genes. The output results of 22 infiltrating immune cells were integrated, and the component matrix of immune cells was generated for analysis. Using the results of immune infiltration analysis of the above shared markers, the correlation between core markers and the expression of immune infiltrating cells was analyzed by non-parametric correlations (spearman) method.

## 3 Results

### 3.1 Differential genetic screening

**Figure 1:**
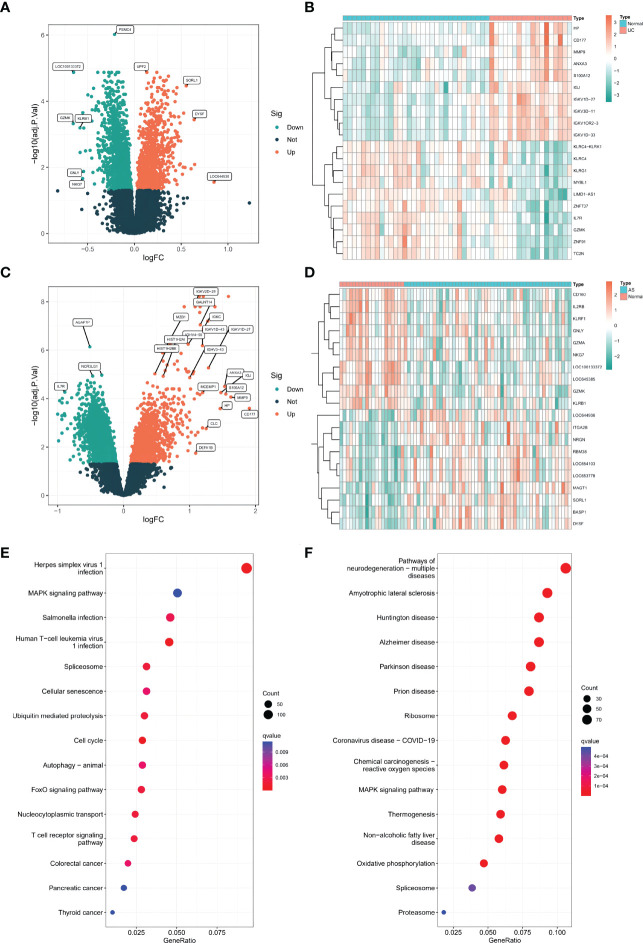
**(A)** The volcano plots of DEGs in GSE126124(n=50, p < 0.05). **(B)** The heatmap plots of DEGs in GSE126124. **(C)** The volcano plots of DEGs in GSE73754 (n=68, p < 0.05). **(D)** The heatmap plots of DEGs in GSE73754. **(E)** The GO term analysis of DEGs in GSE126124. **(F)** The GO term analysis of DEGs in GSE73754.

A total of 4428 differentially expressed genes (DEGs) were screened between UC patients and normal controls, with 1912 upregulated and 2516 downregulated DEGs shown in volcano plots (**Figure 1A**). Of these DEGs, the top 20 differentially expressed genes are displayed in heatmaps (**Figure 1B**). Additionally, 2438 DEGs were identified between AS and normal controls, including 1037 upregulated genes and 1401 downregulated genes (**Figure 1C**). The top 20 genes are listed in **Figure 1D**. KEGG pathway enrichment analysis was used to analyze the DEGs. Interestingly, KEGG analysis showed that both AS and UC were enriched in the mitogen-activated protein kinase (MAPK) pathway (**Figures 1E, F**).

### 3.2 Construction and module analysis of weighted gene co-expression network (WGCNA)

**Figure 2:**
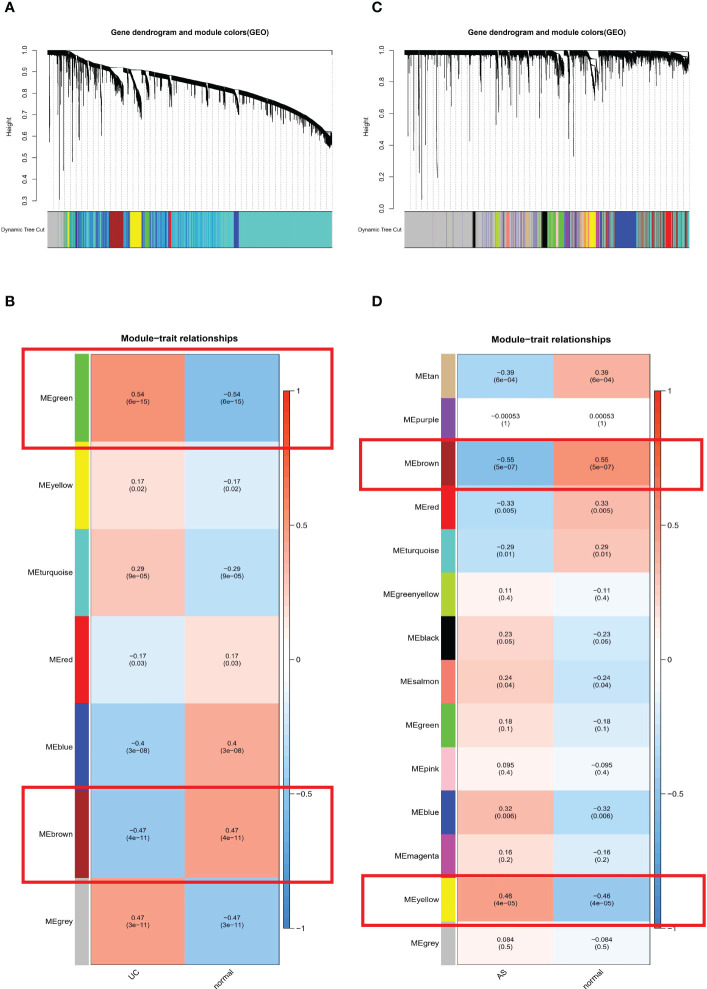
**(A)** The cluster dendrogram of co-expression in UC. **(B)** Correlation between modules and clinical traits in UC. **(C)** The cluster dendrogram of co-expression in AS. **(D)** Correlation between modules and clinical traits in AS.

WGCNA was used to identify clusters of co-expressed genes whose expression differed between UC and AS, and the correlation of combined modules with disease characterization was calculated. It was determined that a soft threshold power of β=35 was set in the UC model, and the soft threshold power in the AS modeling set was 15 (**Figures 2A, C**). After merging similar gene modules, seven modules were identified in the UC model set and 14 modules in the AS model set. As shown in **Figure 2B**, the brown module had the strongest positive correlation with the occurrence of UC (r=0.47) and the green module had the strongest negative correlation with the occurrence of UC (r=-0.54). Further, in the AS modeling set, the yellow module in the AS modeling set had the strongest positive correlation with AS occurrence (r=0.46), and the brown module had the strongest negative correlation with AS occurrence (r=-0.55) (**Figure 2D**).

### 3.3 Identification of shared genes and shared pathways

**Figure 3:**
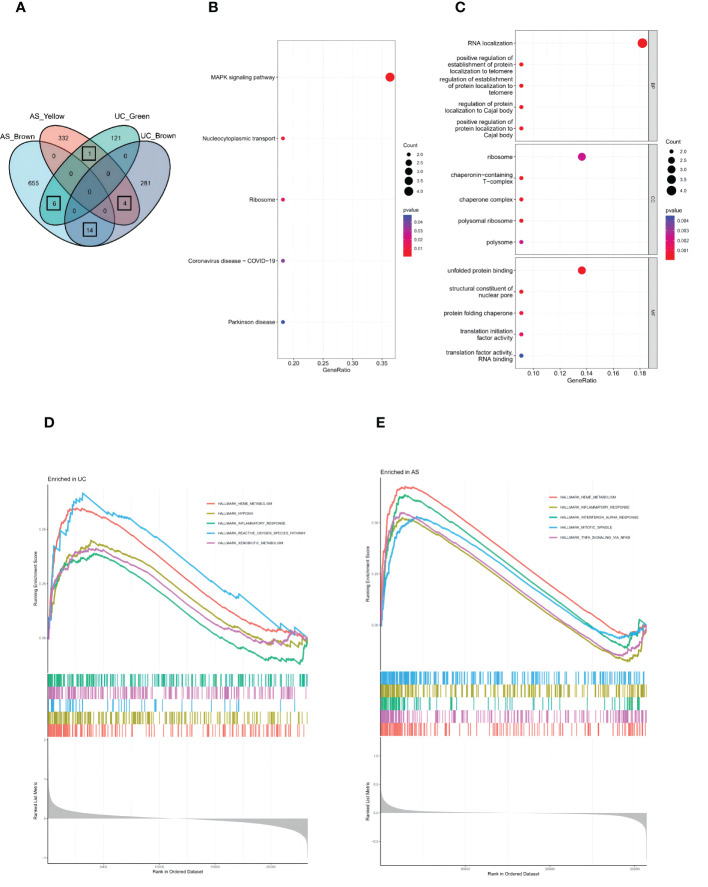
**(A)** The Venn diagram shows an overlap of 25 genes in modules between UC and AS. **(B)** The GO term analysis of 25 shared genes between UC and AS screened by WGCNA. **(C)** The KEGG analysis of 25 shared genes between UC and AS screened by WGCNA. **(D)** Single-gene GSEA analyses result for DEGs of UC. **(E)** Single-gene GSEA analyses result for DEGs of AS.

A total of 25 genes overlapped in the strongest positive and negative modules of UC and AS (**Figure 3A**). These genes may be related to the pathogenesis of UC and AS. Enrichment analysis was performed again for these 25 genes. KEGG analysis showed that these genes may be involved in the MAPK signaling pathway and other pathways (**Figure 3B**). In addition, GO analysis showed that these genes were significantly enriched in RNA localization (**Figure 3C**). Subsequently, we performed GSEA on AS and UC samples and found that inflammatory responses were involved in a common pathogenic process (**Figures 3D, E**). Therefore, we propose a bold conjecture that the occurrence of AS and UC may be jointly driven by the MAPK pathway and immune response.

### 3.4 Identification and validation of potential shared diagnostic markers

**Figure 4:**
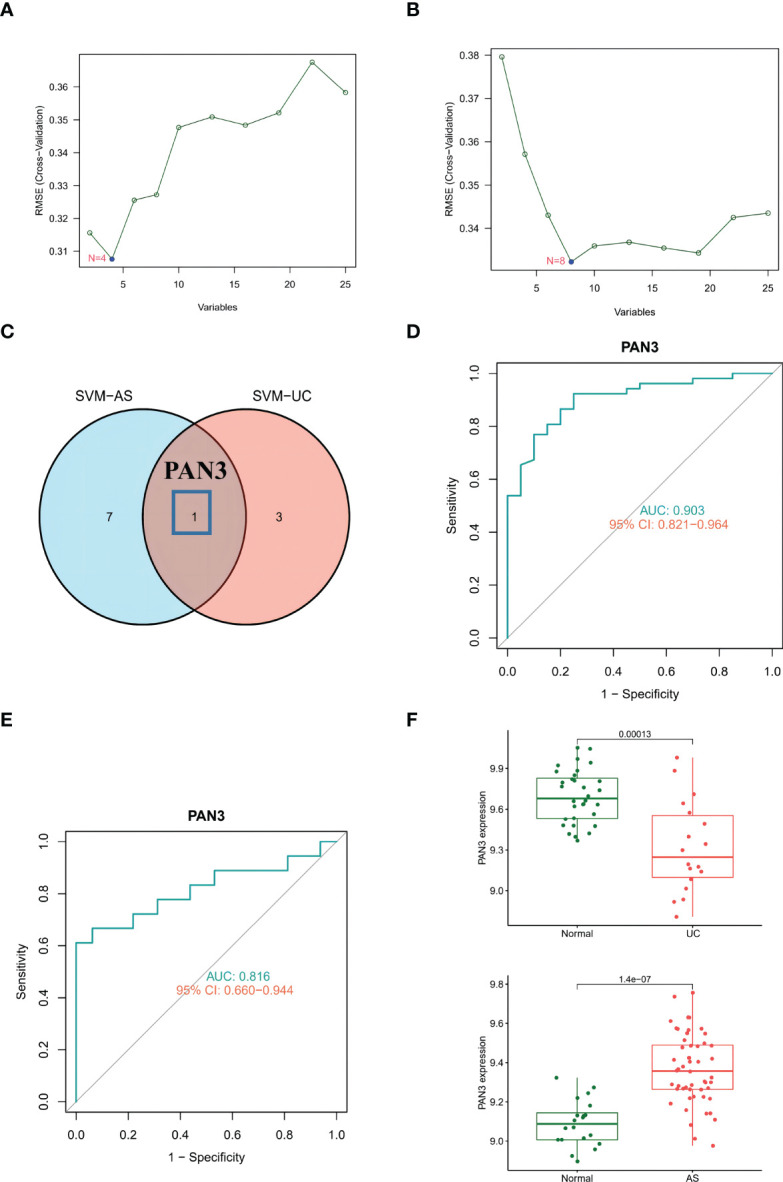
**(A)** SVM-RFE to screen diagnostic markers in GSE126124. **(B)** SVM-RFE to screen diagnostic markers in GSE73754. **(C)** The Venn diagram shows the diagnostic biomarkers between UC and AS. **(D)** ROC curve evaluating the PAN3 diagnostic efficacy in GSE126124. **(E)** ROC curve evaluating the PAN3 diagnostic efficacy in GSE73754. **(F)** PAN3 showed significant differences in GSE126124 and GSE73754 (P<0.05).

SVM-REF is a machine learning method based on a support vector machine. The optimal core gene is found by deleting the feature vector generated by the support vector machine. Based on the 25 shared genes, four possible diagnostic markers were identified in GSE126124 (**Figure 4A**), and eight possible diagnostic markers were identified in GSE73754 (**Figure 4B**). Ultimately, we determined that *PAN3* may be the best diagnostic biomarker for the development of UC and AS (**Figure 4C**). We further identified the diagnostic power of the shared markers based on ROC analysis. In GSE126124, we obtained an AUC of 0.903 (**Figure 4D**). Additionally, the same ROC analysis was performed again for GSE73754, and the result showed that the AUC was 0.816 (**Figure 4E**). Interestingly, differential expression analysis showed that *PAN3* was significantly overexpressed in AS, but expression significantly decreased in UC (**Figure 4F**).

### 3.5 The confirmation of the diagnostic marker gene

**Figure 5:**
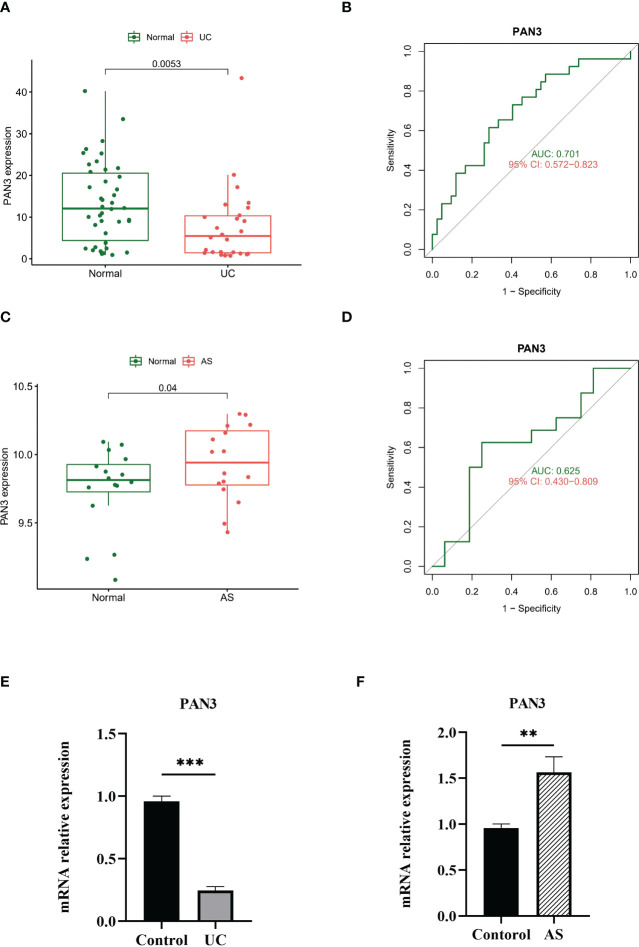
**(A)** PAN3 showed significant differences in GSE3365. **(B)** ROC curve evaluating the PAN3 diagnostic efficacy in GSE3365. **(C)** PAN3 showed significant differences in GSE25101. **(D)** ROC curve evaluating the PAN3 diagnostic efficacy in GSE25101. **(E–F)** Real-time PCR analysis for the mRNA expression levels of PAN3 in PBMC from patients and healthy controls. **P < 0.01; ***P < 0.001.

We verified the diagnostic efficacy of *PAN3* in an independent UC dataset GSE3365. The differential expression analysis showed a significant difference in *PAN3*, similar to the results reported above (**Figure 5A**), AUC= 0.701 (**Figure 5B**). In another independent AS validation set GSE25101, the expression levels of *PAN3* in different samples were significantly different (**Figure 5C**), and the AUC was 0.625 (**Figure 5D**). Additionally, we collected fresh full blood samples from nine patients (3 UC, 3 AS, 3 Controls), extracted PBMC and performed quantitative qPCR analysis to further verify the differential expression of PAN3 in the patient samples. The results showed that PAN3 expression was decreased in UC patients and increased in AS patients compared to normal subjects (**Figures 5E, F**), suggesting that PAN3 has the potential to be a genetic diagnostic marker for both diseases.

### 3.6 Immune infiltration analysis of shared markers

**Figure 6:**
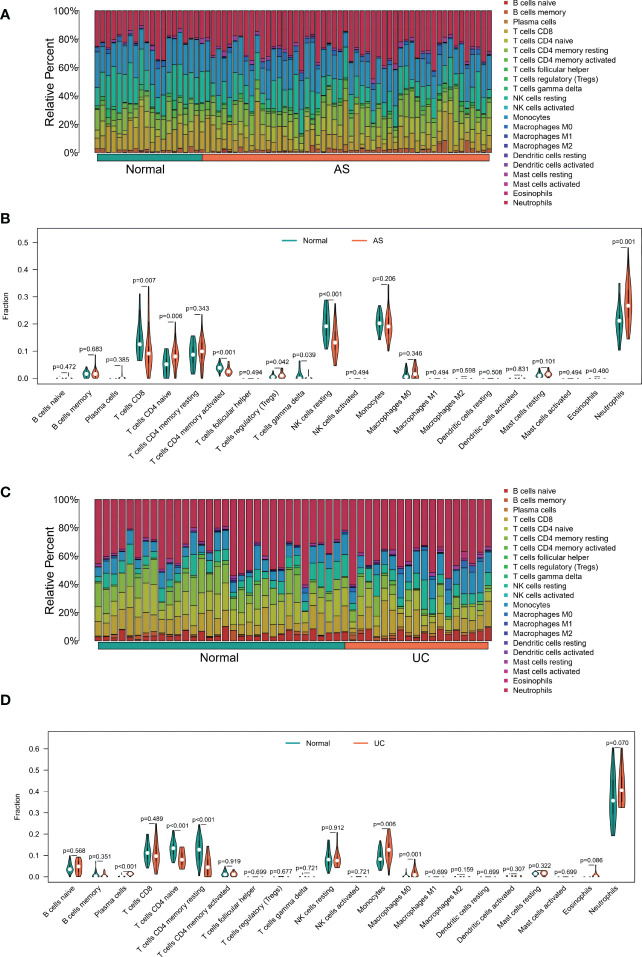
**(A)** The Bar plot of immune cell infiltration between AS and normal. **(B)** The Violin plot of the 22 types of immune cells proportions between AS and normal. **(A, C)** The Bar plot of immune cell infiltration between UC and normal. **(D)** The Violin plot of the 22 types of immune cells proportions between UC and normal.

Considering the important roles of immune and inflammatory responses in the development of UC and joint involvement of AS, we used the CIBERSORT algorithm in enrichment analysis. This enabled analysis of the content of immune cells in different samples. The histograms showed that the proportion of B cell populations in the AS samples was significantly different from that in the UC samples (**Figures 6A, C**). In addition, compared with normal tissues, AS samples had higher levels of CD8 T cells, CD4 naive T cells, CD4 memory activated T cells, regulatory T cells (Tregs), gamma delta T cells, resting NK cells, and neutrophils (**Figure 6B**). However, comparison of UC and normal tissues showed differences in the contents of plasma cells including CD4 naïve T cells, CD4 memory T cells, resting monocytes, and macrophages (M0) (**Figure 6D**). Interestingly, there were similarities in the differences in T cell subsets between the two diseases.

### 3.7 Correlation between shared markers and immune cell content

**Figure 7:**
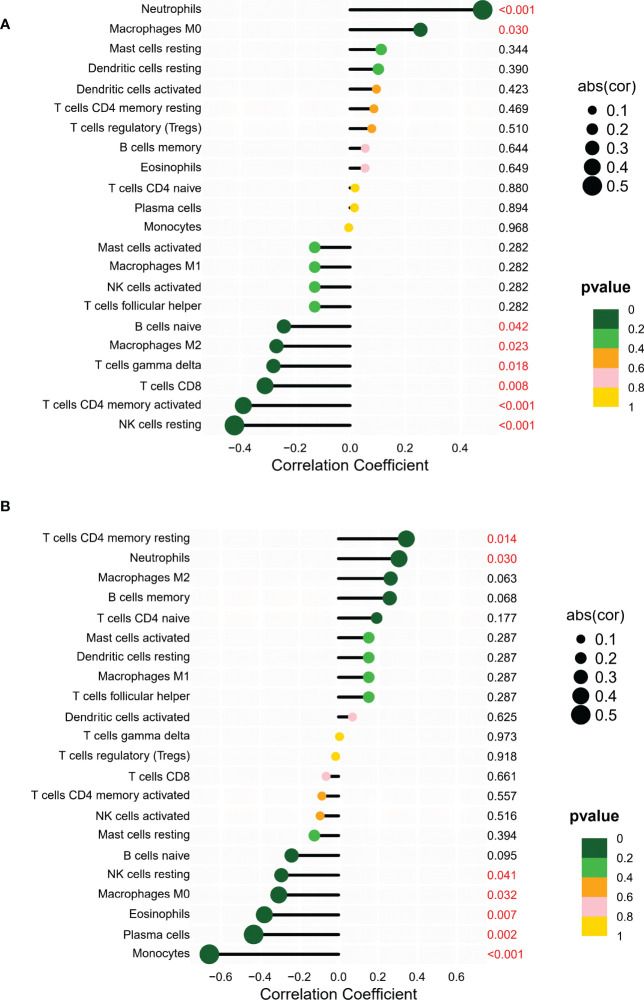
**(A)** Correlation analysis between PAN3 and infiltrating immune cells in AS samples where red represented the positive correlation with a significant difference. **(B)** Correlation analysis between PAN3 and infiltrating immune cells in UC samples where red represented the positive correlation with a significant difference.

The correlation between shared markers and immune cell content was also explored. In AS samples, *PAN3* expression was significantly correlated with naive B cells, CD8 T cells, CD4 memory T cells, gamma delta T cells, resting NK cells, M0s, polarizing macrophages (M2), and neutrophils (**Figure 7A**). In UC samples, *PAN3* was significantly associated with plasma cells, CD4 memory resting T cells, resting NK cells, monocytes, macrophages, eosinophils, and neutrophils (**Figure 7B**).

## 4 Discussion

Existing studies have shown a potential link between gut and joint inflammation (12–14). The similar pathogenesis of inflammatory bowel disease and spondylitis (SpA) has led researchers to propose the theory of the gut-joint axis (15). Currently, a causal relationship between SpA and IBD cannot be ascertained. Therefore, identifying common features of these two diseases allows exploration of the causal relationship between them. In 1973, investigators found that HLA-B27 was highly associated with AS contributing to approximately 20.1% of AS heritability (16). However, HLA-B27 is less specific in AS associated with IBD than in idiopathic AS (7). In this study, we explored the common disease pathways and diagnostic markers involved in UC and AS using bioinformatic analysis. Based on the functional analysis of DEGs screened from different datasets, we found that the occurrence of AS and UC is closely related to the MAPK pathway. Gene clustering analysis was used to screen disease-related modules and shared genes, and functional analysis confirmed the important role of the MAPK signaling pathway. We speculated that the MAPK signaling pathway may be involved in the co-pathogenesis of AS and UC. In addition, we screened the optimal core genes based on the SVM-RFE algorithm, and identified *PAN3* as a possible diagnostic marker of UC and AS.

The MAPK signaling pathway is a classic pathway involved in cell growth, differentiation, stress, and inflammatory responses. The MAPK family members can be divided into two groups: classical MAPKs, including ERK1/2, P38, JNK, and ERK5 (17), and atypical MAPKs, consisting of ERK3, ERK4, ERK7, and NLK (18), with independent or overlapping effects. Many preclinical studies have confirmed that MAPK inhibitors have significant efficacy in experimental colitis animal models (19–21). Moreover, these results suggest the MAPK signaling pathway may play an important role in IBD. In addition, it has been shown that levels of all three phosphorylated MAPKs are significantly elevated in the inflamed intestinal mucosa of IBD patients compared to normal controls, and non-inflamed sites in IBD patients (22). Exploration of potential therapeutic agents for UC, showed that many drugs exert their effects by inhibiting MAPK-related pathways. In a study by Zhou et al., mTOR-dependent autophagy was found to be involved in the regulation of active UC through upstream TLR4-MyD88-MAPK signaling and the downstream NF-kB pathway (23). This demonstrates a close relationship between the MAPK signaling pathway and the mTOR-autophagy axis, providing mechanistic evidence for mTOR inhibitors as candidate therapies for IBD. Wang et al. (2019) used methane-rich saline to protect against acetic acid-induced UC by inhibiting the TLR4/NF-KB/MAPK signaling pathway and promoting the IL-10/JAK1/STAT3-mediated anti-inflammatory response (24).

The MAPK signaling pathway is also strongly associated with the development of AS. A study has shown that simulated inflammatory environments can activate MAPK and TLR signaling pathways in mesenchymal stem cells (MSCs) derived from AS patients, causing upregulation of inflammatory gene expression (25). Moreover, in a study by Yong et al. (26) MAPK7 and NDUFS4 were shown to play an important role in AS through the cAMP-mediated signaling pathways; thus these may be potential targets for indomethacin treatment for AS (26). This study also identified MAPK as a potential common pathway involved in the pathogenesis of both AS and UC. Thus, further investigation of the pathogenesis of extra-intestinal joint inflammatory complications in patients with IBD should be performed.

In this study, *PAN3* was found to be a common diagnostic marker involved in the development of UC and AS, with *PAN3* expression being significantly high in patients with AS; though, significantly reduced in patients with UC. Pan3 and Pan2 form a protein complex involved in the deadenylation of the 3’PolyA tail of eukaryotic mRNA to regulate mRNA stability. Two Pan3 cells comprise an asymmetric dimer, providing a scaffold for Pan2. The Pan2 subunit has nucleic acid exonuclease activity, which is necessary for deadenylation; however, effective deadenylation requires the participation of Pan3 (27). However, the mechanism of Pan3 remains unknown. A study published in 2019 showed high expression of immune cell-associated circular RNA circPan3 in mice and human Lgr5+ intestinal stem cells (ISC). Circular RNA (circRNA) circPan3 is a transcript derived from the *PAN3* gene. CircPan3 in Lgr5-GFR^+^ ISCs promotes the self-renewal ability of ISCs through cryptic ILC2-mediated IL-13-IL-13R signaling. The circRNA circPan3 plays an important role in the self-renewal of ISC (28). Though, whether PAN3 plays a role in patients with UC complicated by AS through mediating effects on intestinal stem cells needs to be further explored.

Studies have shown an immune response and inflammatory activity in both UC and AS diseases with multiple immune cell types (29, 30). In our study, different proportions of B cell populations were involved in the AS and UC samples calculated by CIBERSORT. B cells play an important role in intestinal homeostasis in UC. The IgA-dominated B-cell response is an essential part of the intestinal mucosa that maintains homeostasis and defends against pathogens. It has been demonstrated that in patients with UC, the number of B cells is reduced and the functional activity of B (Breg) cells is decreased (31). Targeted therapies targeting the B-cell compartment have been suggested to be potentially promising in the treatment of IBD (32). The role of B cells in the pathogenesis of AS has rarely been studied, but there is growing evidence for the involvement of B cells in the pathogenesis of AS (33). Significantly elevated B cell counts were found in tissue biopsies from patients with AS with persistent inflammatory activity (34). In addition, large ectopic infiltration of CD20^+^ B cells was observed in the bone marrow and fibrous tissue of patients with AS. This evidence suggests the involvement of active B cells in AS pathogenesis. A clinical trial showed that the removal of B cells through rituximab therapy (anti-CD20) could benefit some AS patients who had not received TNF-α therapy (35). Targeted B-cell therapy holds promise as a new research direction for the treatment of patients with UC and AS. In addition to altered B cell content, many T-lymphocyte immune functions were altered in patients with UC and AS in this study. An imbalance of CD4^+^ T cell subsets is strongly associated with the development of UC (36). Naive CD4^+^ T cells interacting with dendritic cells carrying homologous antigens can differentiate into different effector cell subtypes, including Treg, Th1, Th2, Th9, Th17, and Th22, each of which produces characteristic cytokines to perform their corresponding biological functions (37). A meta-analysis of peripheral blood lymphocytes from patients with AS by Liu et al., showed no significant difference in the percentage of T cells, NK cells, and NKT cells between AS patients and healthy controls. However, there was a significant increase in B cells (38), which was consistent with the results of our partial findings.

Interestingly, we found that monocytes and neutrophils showed similar trends in UC and AS. In a study by Furukawa et al. (2021), peripheral blood monocyte counts were shown to be independently and negatively associated with clinical remission and mucosal healing (MH) in Japanese UC patients. Peripheral blood mononuclear cell counts could also be used as complementary blood markers for MH in UC patients with low C-reactive protein (CRP) levels (39). Monocytes have also been shown to play a key role in the pathogenesis of SpA. Moreover, monocyte subpopulations were found to be positively correlated with AS disease activity parameters and C-reactive protein levels, and monocytes in AS patients significantly increased phagocytic activity than controls (40). Recent evidence has shown that NETs are involved in the pathogenesis of IBD. NETs are the first immune cells to infiltrate the intestinal barrier, and extensive infiltration of NETs is positively correlated with active UC (41). Jiang et al. reported that the upregulation of neutrophils may be a key factor in the progression of AS (42). Interestingly, most of the evidence accumulated thus far points to adaptive immunity being related to AS pathogenesis. However, we have provided evidence to further explore the involvement of innate immunity in the pathogenesis of AS, through exploring the common pathways and diagnostic markers of UC and AS. The question of whether AS is predominantly autoinflammatory or autoimmune is still being explored by researchers (43). However, it is indisputable that our study has some limitations, as only lymphocytes in peripheral blood were studied. As such, we cannot provide a comprehensive response to the inflammatory status of UC and AS.

## 5 Conclusion

**Figure 8:**
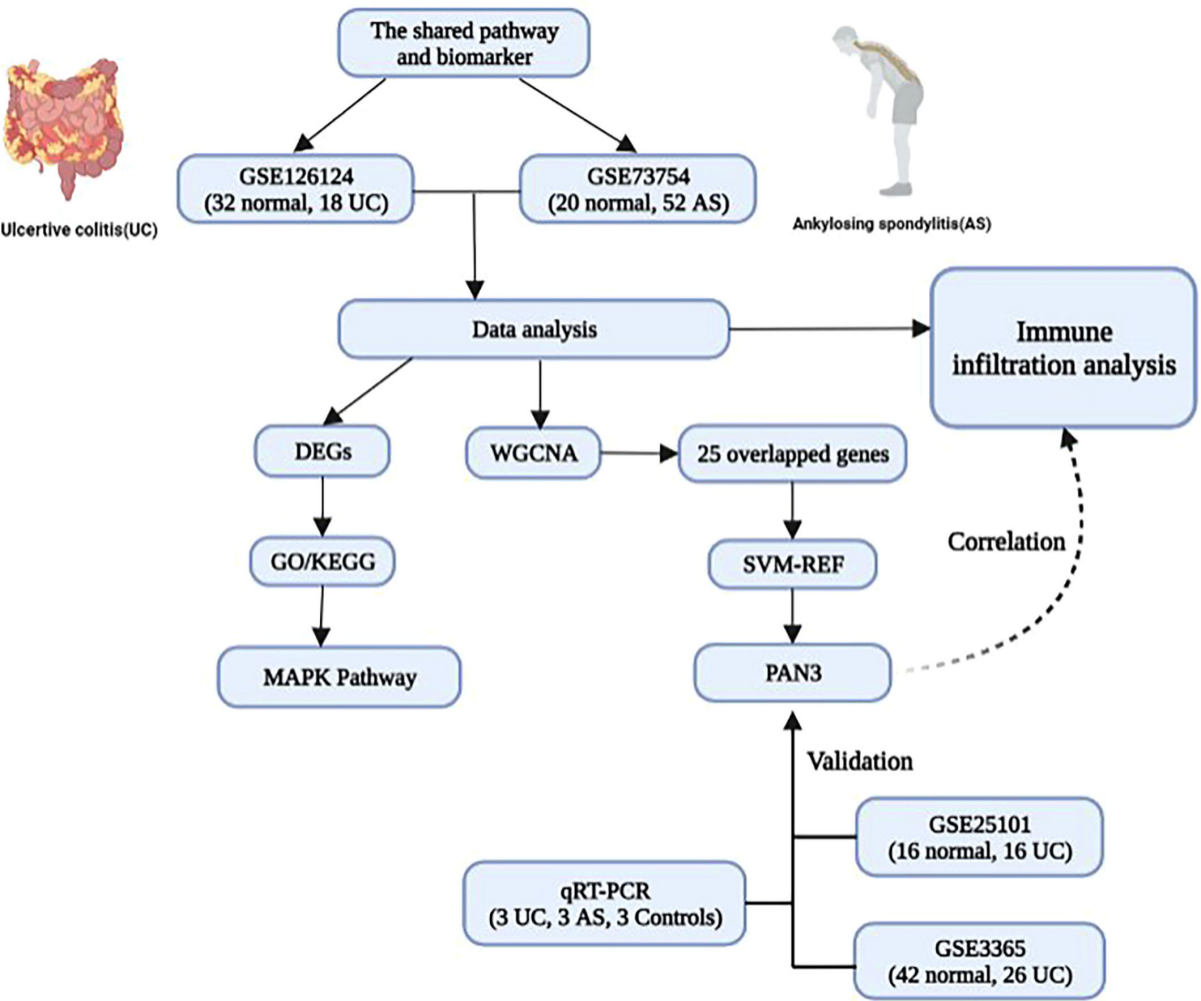
The model diagram of the shared pathway and common biomarker analysis in UC and AS.

This is the first study to use bioinformatic analysis to explore the common pathways and genetic diagnostic markers involved in UC and AS (**Figure 8**). Results suggest that the MAPK signaling pathway may be associated with the pathogenesis of both AS and UC. Moreover, *PAN*3 may be a potential diagnostic marker for patients with UC complicated by AS. Additionally, immune infiltration correlation analysis suggests the pathogenesis of UC and AS may be closely related to innate immune imbalances. This study provides a new perspective for investigating the possible mechanism of AS secondary to UC. In future studies, we will further investigate the mechanism of the MPAK pathway and PAN3 expression changes in conjunction with relevant *in vitro* and Vivo experiments.

## Data availability statement

The datasets presented in this study can be found in online repositories. The names of the repository/repositories and accession number(s) can be found in the article/**Supplementary Material**.

## Ethics statement

The studies involving human participants were reviewed and approved by The Ethics Committee of the Affiliated Huai’an No.1 People’s Hospital with Nanjing Medical University. The patients/participants provided their written informed consent to participate in this study. Written informed consent was obtained from the individual(s) for the publication of any potentially identifiable images or data included in this article.

## Author contributions

MZ, JZ, and HW designed the experiment. LH and JW analyzed the data. MZ and JZ wrote and prepared the manuscript. XY and XZ reviewed the manuscript. All authors contributed to the article and approved the submitted version.

## Supplementary material

Supplementary Figure 1 The Venn diagram shows 7494 common genes in four different databases.
