# Supplementary figures and images for: Exploration of the shared pathways and common biomarker PAN3 in ankylosing spondylitis and ulcerative colitis using integrated bioinformatics analysis
Source: Front Immunol. 2023 Jan 18;14:1089622. doi: 10.3389/fimmu.2023.1089622 (PMC9891726; doi:10.3389/fimmu.2023.1089622)

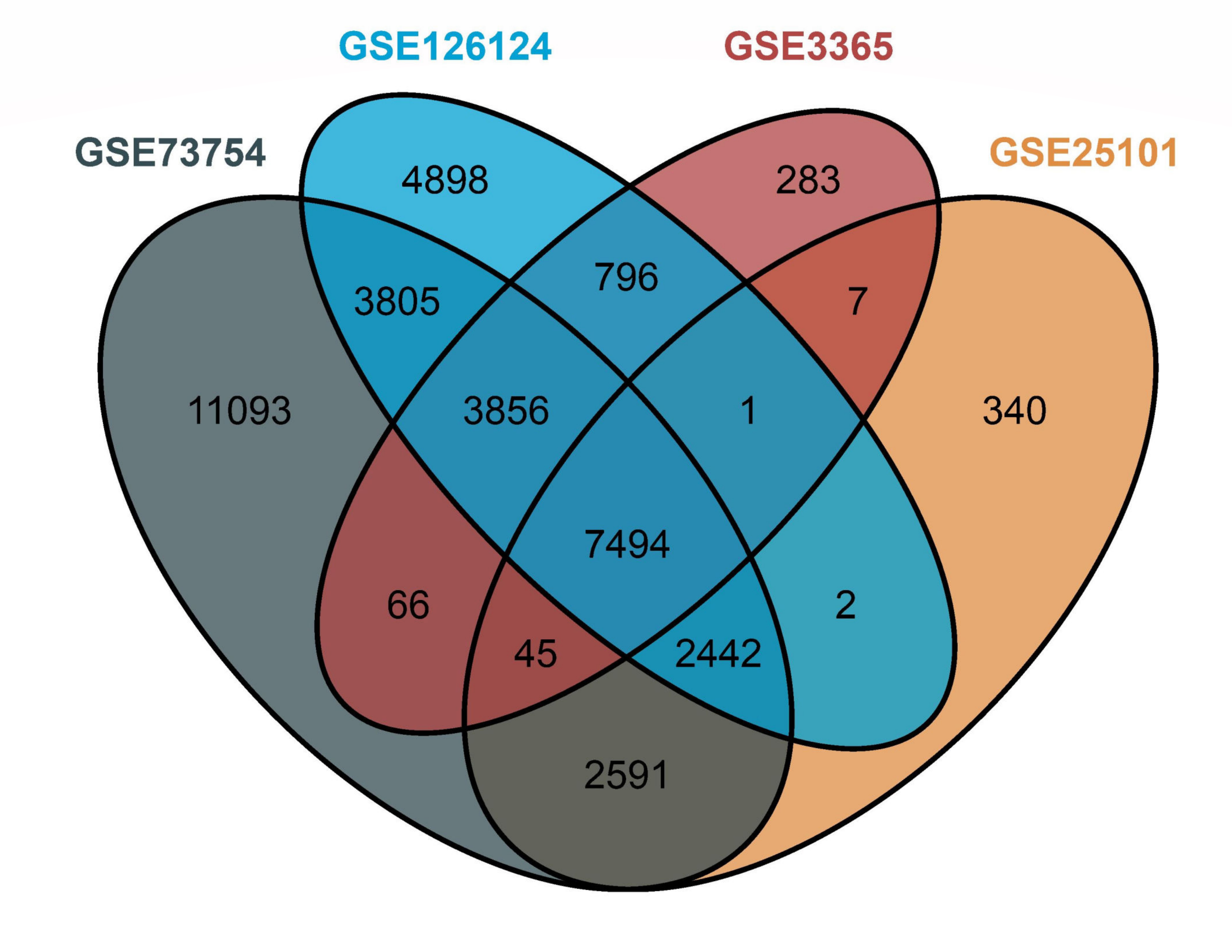

Supplement: Supplementary Figure 1 — The Venn diagram shows 7494 common genes in four different databases. [file Image_1.jpg]
